# Combined neutrophil-lymphocyte ratio and platelet-lymphocyte ratio predicts chemotherapy response and prognosis in patients with advanced gastric cancer

**DOI:** 10.1186/s12885-019-5903-y

**Published:** 2019-07-08

**Authors:** Tetsushi Hirahara, Takaaki Arigami, Shigehiro Yanagita, Daisuke Matsushita, Yasuto Uchikado, Yoshiaki Kita, Shinichiro Mori, Ken Sasaki, Itaru Omoto, Hiroshi Kurahara, Kosei Maemura, Keishi Okubo, Yoshikazu Uenosono, Sumiya Ishigami, Shoji Natsugoe

**Affiliations:** 1 Department of Digestive Surgery, Breast and Thyroid Surgery, Kagoshima University Graduate School of Medical and Dental Sciences, Kagoshima, Japan; 2 Department of Onco-biological Surgery, Kagoshima University Graduate School of Medical and Dental Sciences, 8-35-1 Sakuragaoka, Kagoshima, 890-8520 Japan

**Keywords:** Neutrophil–lymphocyte ratio, Platelet–lymphocyte ratio, Chemotherapy response, Prognosis, Advanced gastric cancer

## Abstract

**Background:**

The neutrophil–lymphocyte ratio (NLR) and platelet–lymphocyte ratio (PLR) are representative blood markers of systemic inflammatory responses. However, the clinical significance of the combination of these markers is unclear. This study aimed to investigate the NLR and PLR in patients with advanced gastric cancer treated with chemotherapy and assess the clinical utility of a new blood score combining the NLR and PLR (NLR-PLR score) as a predictor of tumor response and prognosis.

**Methods:**

We retrospectively analyzed 175 patients with gastric cancer receiving chemotherapy or chemoradiotherapy. These patients were categorized into progressive disease (PD) and non-PD groups according to tumor response. The NLR and PLR before treatment were examined, and the cut-off values were determined. The NLR-PLR score ranged from 0 to 2 as follows: score of 2, high NLR (> 2.461) and high PLR (> 248.4); score of 1, either high NLR or high PLR; score of 0, neither high NLR nor high PLR.

**Results:**

With regard to tumor response, 64 and 111 patients had PD and non-PD, respectively. The NLR-PLR score was significantly higher in patients with PD than in those with non-PD (*p* = 0.0009). The prognosis was significantly poorer in patients with a higher NLR-PLR score than in those with a lower NLR-PLR score (*p* <  0.0001). Multivariate analysis demonstrated that the NLR-PLR score was an independent prognostic factor for prediction of overall survival (*p* = 0.0392).

**Conclusion:**

Low-cost stratification according to the NLR-PLR score might be a promising approach for predicting tumor response and prognosis in patients with advanced gastric cancer.

## Background

Gastric cancer is one of the most common gastrointestinal malignancies and is the third leading cause of cancer-related mortality worldwide [1]. Currently, various therapeutic strategies are available for the clinical management of patients with early gastric cancer having a favorable prognosis. In particular, endoscopic treatments, such as endoscopic submucosal dissection, have been widely accepted as minimally invasive approaches in selected patients with early gastric cancer. On the other hand, in patients with advanced or recurrent gastric cancer, the clinical outcome is poor owing to malignant characteristics. The 5-year survival rates in patients with stage IIIC and IV gastric cancer have been reported to be 20.2 and 8.8%, respectively [2]. The Japanese Gastric Cancer Treatment Guidelines 2014 (ver. 4) have suggested chemotherapy for initial treatment in patients with unresectable or recurrent gastric cancer having a performance status of 0–2 [3]. There has been focus on neoadjuvant chemotherapy (NAC) as a novel therapeutic strategy, and several studies have mentioned that NAC followed by gastrectomy is a promising approach to improve prognosis in patients with locally advanced gastric cancer [4–6]. Recent developments in chemotherapy are worthy of attention, and an improved prognosis is expected even in patients with advanced gastric cancer. However, it is clinically difficult to predict tumor response and prognosis before the initiation of chemotherapy. Thus, there are few prognostic predictors in the clinical management of patients with advanced gastric cancer.

To date, several investigators have demonstrated a close relationship between the systemic inflammatory response and tumor progression in various malignancies, including gastric cancer [7, 8]. The neutrophil–lymphocyte ratio (NLR) and platelet–lymphocyte ratio (PLR) are representative blood markers of the systemic inflammatory response. We have previously reported that preoperative assessment of the NLR status has clinical utility for predicting tumor progression and prognosis in patients with resectable gastric carcinoma and esophageal squamous cell carcinoma [9, 10]. Similarly, recent studies have shown that a high PLR is associated with tumor aggressiveness in patients with several neoplasms, including gastric cancer [11–14]. However, the clinical relevance of a new blood score that combines the NLR and PLR (NLR-PLR score) has not been assessed in patients with gastric cancer.

The purpose of the present study was to investigate the NLR and PLR before chemotherapy or chemoradiotherapy in patients with unresectable advanced and recurrent gastric cancer and to evaluate the relationship between tumor response and NLR/PLR. Furthermore, the study assessed the clinical potential of the NLR-PLR score as a new blood predictor of tumor response and prognosis.

## Methods

### Patients

**Fig. 1:**
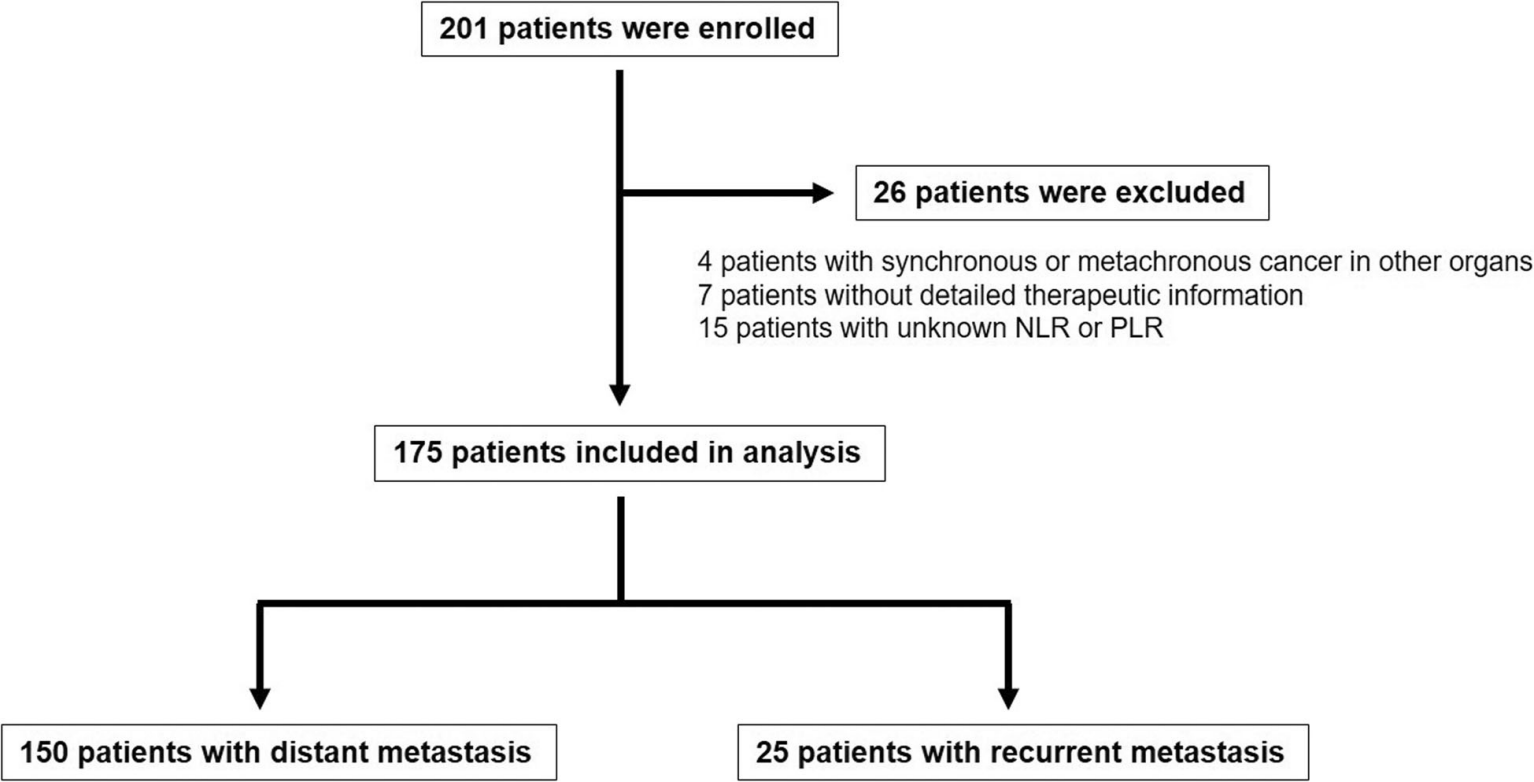
Flowchart of patient selection

The present study retrospectively enrolled 201 patients with unresectable advanced and recurrent gastric cancer who received chemotherapy or chemoradiotherapy at the Kagoshima University Hospital (Kagoshima, Japan) between January 2007 and December 2017. The exclusion criteria were as follows: synchronous or metachronous cancer in other organs (*n* = 4), absence of detailed therapeutic information (*n* = 7), and an unknown NLR or PLR (*n* = 15). Finally, 175 patients (118 men and 57 women; age range, 30–87 years; mean age, 65.8 years) were included in the present study (Fig. 1). Of the 175 patients, 150 and 25 patients had primary gastric tumors with distant metastasis and recurrent metastasis after gastrectomy, respectively. Among 175 patients with unresectable advanced and recurrent gastric cancer, 39 patients had more than 2 distant metastatic sites. Peritoneal dissemination, distant lymph node metastasis, and hematogenous metastasis were noted in 92, 63, and 51 patients, respectively. All patients underwent blood examinations, esophagogastroduodenoscopy, endoscopic ultrasonography, fluoroscopy, and computed tomography before chemotherapy or chemoradiotherapy. Furthermore, 160 patients underwent fluorodeoxyglucose positron emission tomography. Patients were classified and staged according to the tumor–node–metastasis classification for gastric carcinoma established by the International Union Against Cancer [15]. This retrospective observational study was approved by the Ethics Committee of the Kagoshima University (approval number: 28–37).

### Treatment and assessment of tumor response

With regard to chemotherapy, 92 and 79 patients received cisplatin/fluoropyrimidine and paclitaxel/fluoropyrimidine-based chemotherapy as the first-line regimen, respectively. Additionally, 4 patients received cisplatin/fluoropyrimidine-based chemotherapy with concomitant radiation therapy at a total dose of 40–50 Gy.

The clinical responses were assessed after 2 or 3 cycles of chemotherapy or chemoradiotherapy. Tumor response was assessed using the Response Evaluation Criteria in Solid Tumors (RECIST), and it was categorized into progressive disease (PD) and non-PD [16]. Overall survival was calculated from the date of treatment initiation to the date of death or last follow-up.

### Blood analysis for the determination of the NLR and PLR

Blood samples were collected within 1 week before the initiation of chemotherapy or chemoradiotherapy. Neutrophils, lymphocytes, and platelets were counted using an XE-2100 automated hematology analyzer (Sysmex Co., Kobe, Japan). The NLR was determined as the neutrophil count divided by the lymphocyte count, while the PLR was determined as the platelet count divided by the lymphocyte count.

### Statistical analysis

The differences in the associations between tumor response and the NLR or PLR were assessed using the Wilcoxon rank-sum test. Receiver operating characteristic (ROC) curves were constructed, and the areas under the curves (AUCs) were calculated to evaluate the predictive abilities of the NLR and PLR for discriminating patients with PD from those with non-PD. The relationships between tumor response and the NLR-PLR score were assessed using the χ^2^ test. Survival was analyzed using Kaplan–Meier curves, and prognostic differences were examined using the log-rank test. Prognostic factors were assessed using univariate and multivariate analyses (Cox proportional hazard regression model). All statistical analyses were performed using SAS statistical software (SAS Institute Inc., Cary, NC, USA). A *p* value of < 0.05 was considered statistically significant.

## Results

### Tumor response after treatment and additional surgery

According to the RECIST criteria, 64 and 111 patients had PD and non-PD, respectively. Consequently, the disease control rate was 63.4% (111/175). Additional surgery was performed in 2 and 45 patients with PD and non-PD, respectively.

### Relationship between tumor response and NLR/PLR

**Fig. 2:**
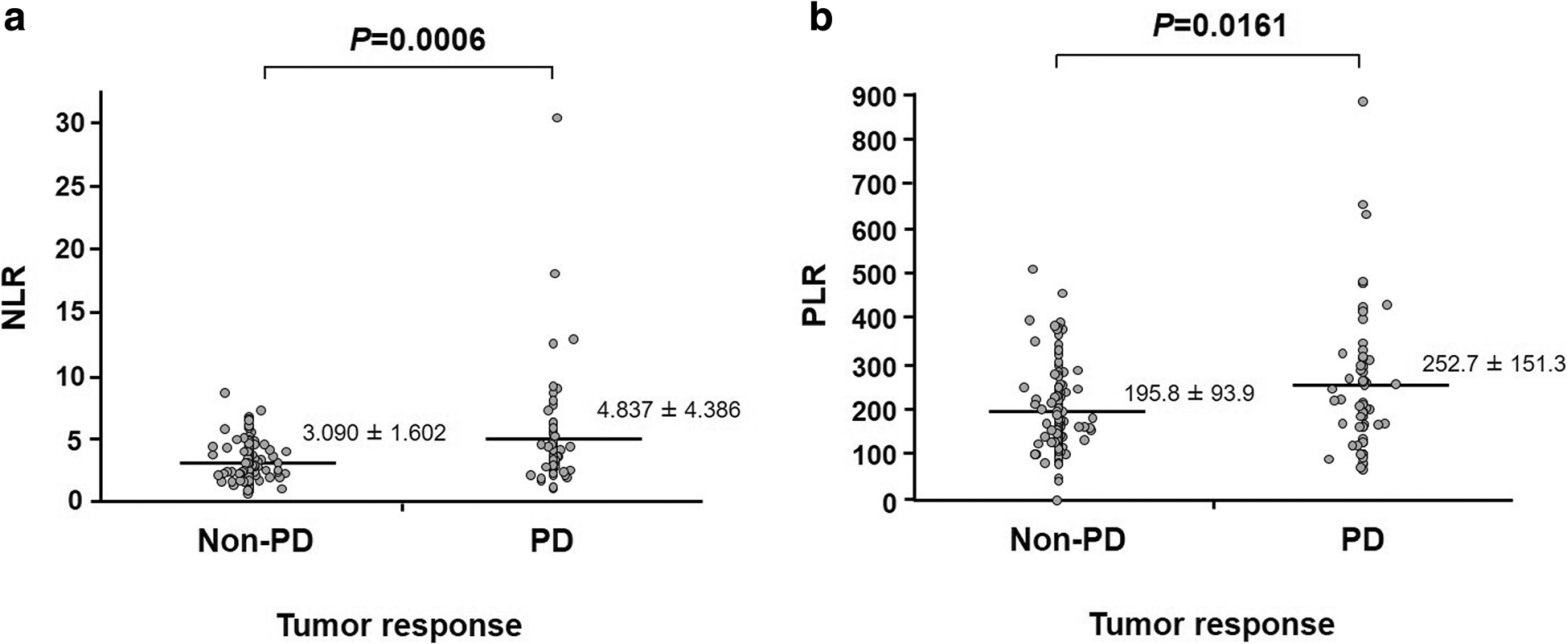
Relationship between tumor response and the NLR (**a**)/PLR (**b**). Horizontal bars indicate mean values of the NLR and PLR

Among the 175 patients, the NLR ranged from 0.534 to 30.333. The mean (± SD) NLR in the 64 and 111 patients with PD and non-PD were 4.837 ± 4.386 and 3.090 ± 1.602, respectively (Fig. 2a). The NLR was significantly higher in patients with PD than in those with non-PD (*p* = 0.0006).

The PLR ranged from 1.2 to 873.3. The mean (± SD) PLRs in the patients with PD and non-PD were 252.7 ± 151.3 and 195.8 ± 93.9, respectively (Fig. 2b). The PLR was significantly higher in patients with PD than in those with non-PD (*p* = 0.0161).

**Fig. 3:**
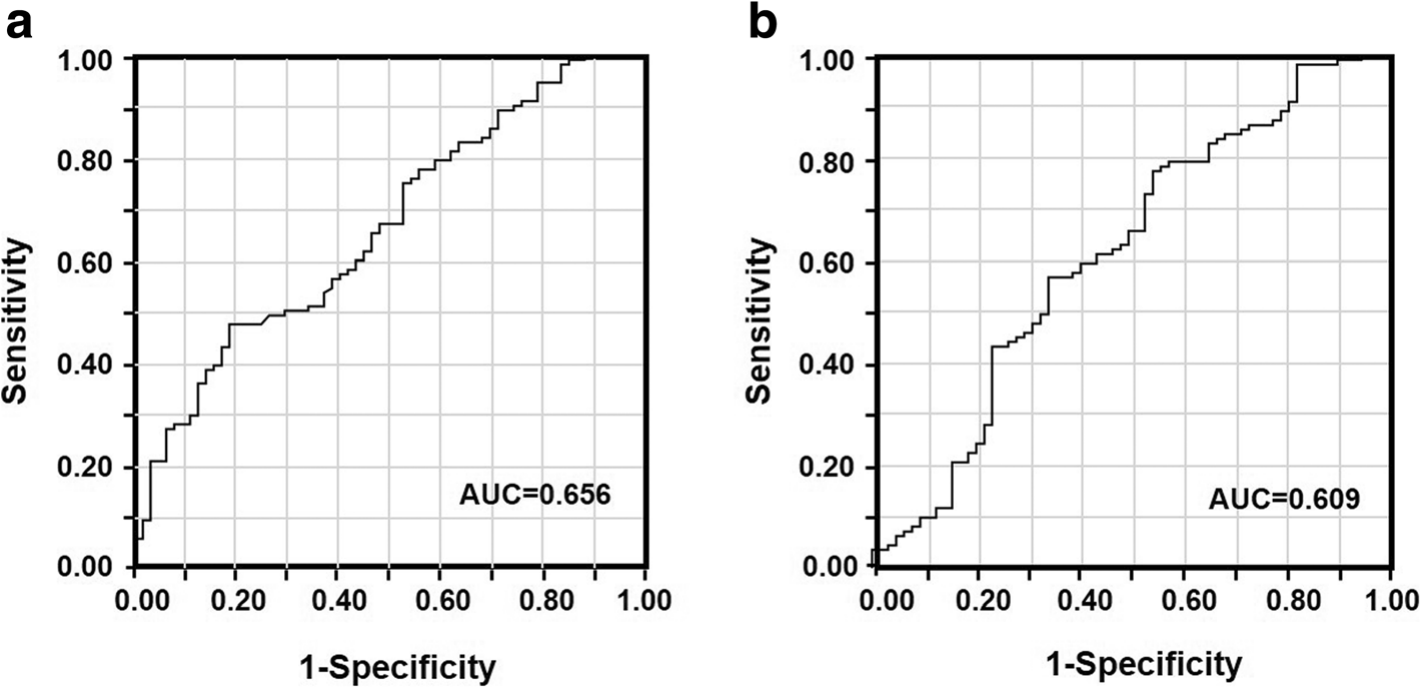
Receiver operating characteristic curves for discriminating patients with PD and those with non-PD according to values of the neutrophil–lymphocyte ratio (**a**) and platelet–lymphocyte ratio (**b**)

In ROC analysis, the AUCs for discriminating patients with PD from those with non-PD according to the NLR and PLR were 0.656 and 0.609, respectively (Fig. 3a and b). According to the findings of the ROC analysis, the cut-off values for the NLR and PLR were set at 2.461 and 248.4, respectively. The sensitivity and specificity for the NLR were 0.469 and 0.813, respectively, while the sensitivity and specificity for the PLR were 0.775 and 0.453, respectively. The patients were divided into the following groups according to the cut-off values of the NLR and PLR: high (> 2.461; *n* = 107) and low NLR status (≤ 2.461; *n* = 68) or high (> 248.4; *n* = 55) and low PLR status (≤ 248.4; *n* = 120). This binary system was used to determine the NLR-PLR score.

### Relationship between tumor response and the NLR-PLR score

The NLR-PLR score ranged from 0 to 2 as follows: score of 2, high NLR (> 2.461) and high PLR (> 248.4); score of 1, either high NLR or high PLR; score of 0, neither high NLR nor high PLR.

NLR-PLR scores of 0, 1, and 2 were noted in 60 (34.3%), 68 (38.9%), and 47 (26.9%) patients, respectively. The NLR-PLR score was significantly higher in patients with PD than in those with non-PD (*p* = 0.0009) (Table 1).

**Table 1:** Relationship between tumor response and the NLR-PLR score

	NLR-PLR score (%)			
Tumor response	0 (*n* = 60)	1 (*n* = 68)	2 (*n* = 47)	*p* value
PD (*n* = 64)	11 (17.2)	29 (45.3)	24 (37.5)	0.0009
Non-PD (*n* = 111)	49 (44.1)	39 (35.1)	23 (20.7)	

*NLR* neutrophil–lymphocyte ratio, *PD* progressive disease, *PLR* platelet–lymphocyte ratio

### Relationship between prognosis and the NLR-PLR score

**Fig. 4:**
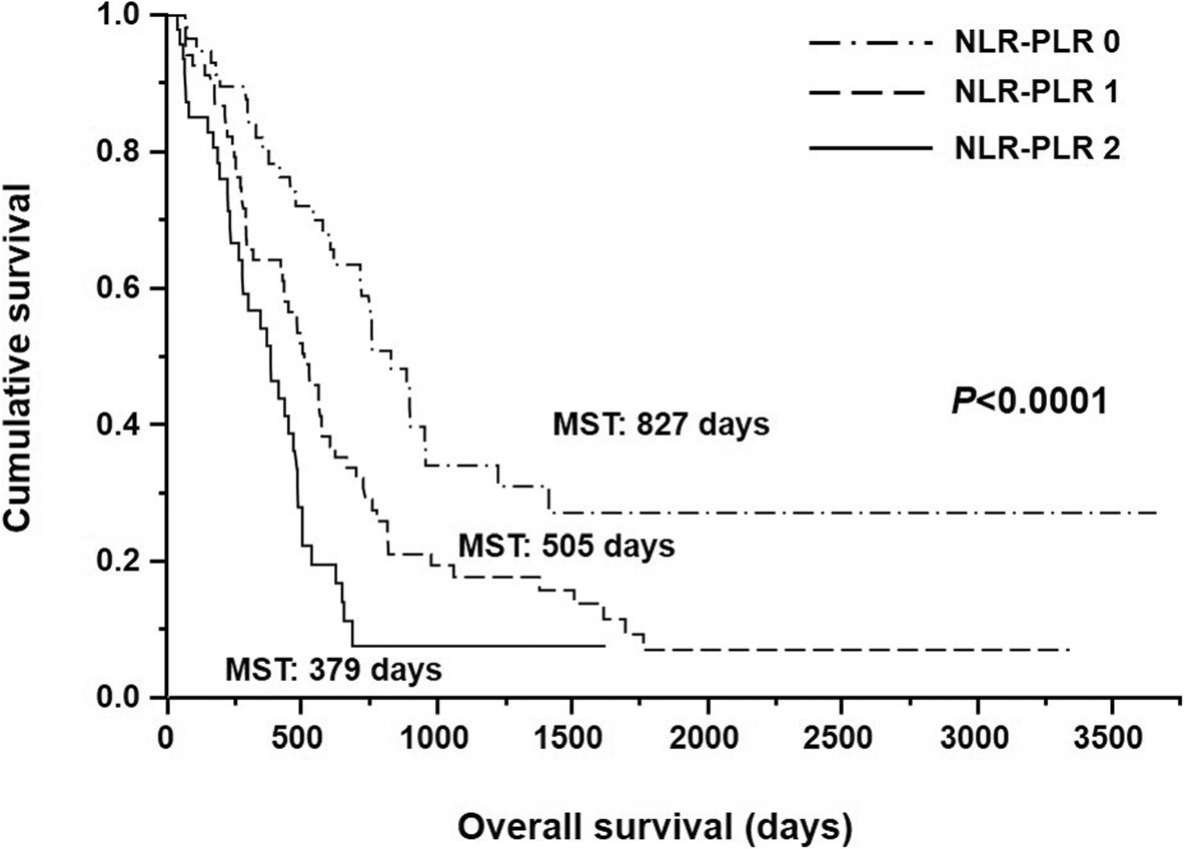
Kaplan–Meier survival curves according to the NLR-PLR score. Survival is significantly poorer in patients with a high NLR-PLR score than in those with a low NLR-PLR score (*p* <  0.0001)

The median survival durations in patients with NLR-PLR scores of 0, 1, and 2 were 827, 505, and 379 days, respectively (Fig. 4). Overall survival differences according to the NLR-PLR score were found to be significant (*p* <  0.0001).

Univariate analysis indicated that therapeutic type, tumor response, and NLR-PLR score were significantly associated with overall survival (*p* = 0.0227, *p* <  0.0001, and *p* <  0.0001, respectively) (Table 2). Multivariate analysis showed that tumor response and NLR-PLR score were independent prognostic factors (*p* <  0.0001 and *p* = 0.0392, respectively) (Table 2).

**Table 2:** Univariate and multivariate analyses for survival

Independent factor	Univariate analysis			Multivariate analysis		
	Hazard ratio	95% CI	*p* value	Hazard ratio	95% CI	*p* value
Sex			0.7321			
Female	1.000	reference				
Male	1.066	0.742–1.557				
Age (years)			0.2095			
< 70	1.000	reference				
≥ 70	1.257	0.878–1.786				
Therapeutic type			0.0227			0.1226
Chemoradiotherapy	1.000	reference		1.000	reference	
Chemotherapy	5.369	1.199–94.547		3.499	0.770–61.865	
Tumor response			< 0.0001			< 0.0001
Non-PD	1.000	reference		1.000	reference	
PD	5.145	3.523–7.522		4.226	2.834–6.328	
NLR-PLR score			< 0.0001			0.0392
0	1.000	reference		1.000	reference	
1	1.923	1.259–2.991	0.0023	1.363	0.875–2.157	0.1726
2	3.296	2.027–5.390	< 0.0001	1.958	1.167–3.299	0.0110

*CI* confidence interval, *NLR* neutrophil–lymphocyte ratio, *PD* progressive disease, *PLR* platelet–lymphocyte ratio

## Discussion

Most previous studies have independently investigated the NLR and PLR and have assessed the clinical significance of these blood markers in patients with various malignancies, including gastric cancer [12, 17–22]. However, we combined the NLR and PLR and created the NLR-PLR score as a new scoring system for predicting tumor response and prognosis in patients with advanced or recurrent gastric cancer receiving chemotherapy. To the best of our knowledge, this is the first study to determine the clinical value of the NLR-PLR score in patients with gastric cancer.

Tumor response is one of the most important prognostic factors in patients with unresectable advanced or recurrent gastric cancer treated with chemotherapy or chemoradiotherapy. In the present study, the median survival rates of patients with PD and non-PD were 267 and 754 days, respectively (data not shown). This finding indicates the therapeutic requirement to distinguish responders from non-responders. Unfortunately, it is clinically difficult to predict tumor response using clinicopathological information before treatment. Therefore, we focused on the NLR and PLR to overcome issues with prediction. The NLR and PLR are well-known prognostic markers associated with the systemic inflammatory response, and the immune environment of the host has a great influence on these blood markers [8, 23]. Initially, we examined the relationship between tumor response and the NLR/PLR to assess their clinical potential as strategic blood markers in the management of patients with advanced gastric cancer. The present study demonstrated a close association between PD and a high NLR/PLR. Wang et al. assessed 120 patients with unresectable gastric cancer and reported that patients with a high baseline NLR/PLR had a significantly decreased response to chemotherapy [19]. These findings suggest that the NLR and PLR are candidate blood markers for discriminating between responders and non-responders among patients with unresectable gastric cancer.

In this study, we proposed the NLR-PLR score as a promising prognostic predictor. Surprisingly, the NLR-PLR score was significantly associated with the tumor response to chemotherapy or chemoradiotherapy. Specifically, the NLR-PLR score was 2 in 24 of 64 patients (37.5%) with PD and 0 in 49 of 111 patients (44.1%) with non-PD. Moreover, a NLR-PLR score of 1 or 2 was common among patients with PD (82.8%). These results indicate that the NLR-PLR score is clinically useful as a novel combined blood predictor of tumor response to first-line chemotherapy. Tumor cells produce cancer-related inflammatory mediators, such as tumor necrosis factor-α, interleukin-3 (IL-3), and IL-6 [24]. Next, these inflammatory response can result in a relative neutrophilia, thrombocytosis, and lymphocytopenia. Finally, these phenomenon causes elevated NLR and PLR [9, 10]. Accordingly, high NLR-PLR score is associated with tumor aggressiveness. Since patients with high malignant behaviors have a tendency to chemoresistance [25], this study may indicate a close relationship between NLR-PLR score and tumor response to chemotherapy.

We also evaluated the relation between the NLR-PLR score and prognosis in the same population. Kaplan–Meier analysis showed that the median survival duration was greater in patients with an NLR-PLR score of 0 than in those with an NLR-PLR score of 1 or 2. Accordingly, chemosensitivity might be higher in patients with an NLR-PLR score of 0 than in those with an NLR-PLR score of 1 or 2. Moreover, the median survival durations in patients with NLR-PLR score of 0, low NLR, and low PLR were 827, 750, and 619 days, respectively (data not shown). These results may suggest that the NLR-PLR scoring system can discriminate patients with better prognosis after chemotherapy from all patients, compared with NLR or PLR alone. This would be the greatest advantage of the NLR-PLR score. NLR-PLR score and tumor response were identified as independent prognostic factors for prediction of overall survival in multivariate analysis. As tumor response is unknown before treatment, the NLR-PLR score is a potentially useful prognostic predictor that can be assessed before treatment. Therefore, the NLR-PLR score can help in the selection of patients who need chemotherapy or chemoradiotherapy for the clinical management of advanced gastric cancer. The NLR-PLR score can be easily determined by calculating the NLR and PLR with a small volume of blood (only 2 ml). Thus, assessment of the NLR-PLR score is inexpensive.

The present study had several limitations. This preliminary study involved a retrospective analysis in a small population (*n* = 175) from a single institution. These limitations may have resulted in bias that might have influenced several study results. Consequently, larger validation studies are needed to confirm our findings. Currently, we are planning a further study to assess the clinical utility of the NLR-PLR score in patients with other malignancies, such as esophageal, hepatocellular, pancreatic, and colorectal cancer.

## Conclusions

We demonstrated that the NLR-PLR score is a useful blood marker for predicting therapeutic responses to chemotherapy or chemoradiotherapy and survival outcomes in patients with unresectable advanced and recurrent gastric cancer. In the near future, we believe that the NLR-PLR scoring system will help in the decision-making of therapeutic strategies as a key marker in the clinical management of patients with advanced gastric cancer.

## Data Availability

The datasets used and/or analyzed during the current study are available from the corresponding author on reasonable request.

## Abbreviations

AUCs: Areas under the curves
CI: Confidence interval
NAC: Neoadjuvant chemotherapy
NLR: Neutrophil–lymphocyte ratio
PD: Progressive disease
PLR: Platelet–lymphocyte ratio
RECIST: Response Evaluation Criteria in Solid Tumors
ROC: Receiver operating characteristic
